# First report of *Metarhizium anisopliae *IP 46 pathogenicity in adult *Anopheles gambiae *s.s. and *An. arabiensis *(Diptera; Culicidae)

**DOI:** 10.1186/1756-3305-2-59

**Published:** 2009-12-01

**Authors:** Ladslaus L Mnyone, Tanya L Russell, Issa N Lyimo, Dickson W Lwetoijera, Matthew J Kirby, Christian Luz

**Affiliations:** 1 Biomedical and Environmental Group, Ifakara Health Institute, PO Box 53, Off Mlabani Passage, Ifakara, Tanzania; 2 Laboratory of Entomology, Wageningen University & Research Centre, PO Box 8031, 6700 EH, Wageningen, the Netherlands; 3 Pest Management Center, Sokoine University of Agriculture, PO Box 3110, Morogoro, Tanzania; 4 Faculty of Biomedical and Life Sciences, University of Glasgow, 120 University Place, G12 8TA, Glasgow, UK; 5 Instituto de Patologia Tropical e Saúde Pública, Universidade Federal de Goiás, CP 131, 74001-970 Goiânia, GO, Brasil; 6 Department of Zoology and Marine Biology, University of Dar es Salaam, PO Box 35064, Dar es Salaam, Tanzania; 7 Vector Group, Liverpool School of Tropical Medicine, Liverpool, L3 5QA, UK

## Abstract

The entomopathogenic fungus *Metarhizium anisopliae *isolate IP 46, originating from a soil sample collected in 2001 in the Cerrado of Central Brazil, was tested for its ability to reduce the survival of adult male and female *Anopheles gambiae *s.s. and *An. arabiensis *mosquitoes. A 6-h exposure to the fungus coated on test paper at a concentration of 3.3 × 10^6 ^conidia cm^-2 ^reduced the daily survival of both mosquito species (HR = 3.14, *p *< 0.001), with higher risk of dying in *An. gambiae *s.s relative to *An. arabiensis *(HR = 1.38, *p *< 0.001). Fungal sporulation was observed in >95% of mosquito cadavers in the treatment groups. The results indicate that *M. anisopliae *IP 46 has the potential to be a bio-control agent for African malaria vector species, and is a suitable candidate for further research and development.

## Findings

*Metarhizium anisopliae *IP 46 has shown ovicidal effects against the eggs of *Aedes *spp in Brazil [1-3]. However, its pathogenicity against adult malaria vectors has never been explored. As such, we examined the effect of this strain against laboratory-reared adult *Anopheles gambiae sensu stricto *and *An. arabiensis*, with the aim to include IP 46 in the spectrum of fungal candidates available for use as bio-control agents.

The fungus was imported as conidia from the Institute of Tropical Pathology and Public Health, Federal University of Goiás, Goiânia, Brazil (Tropical Pesticides Research Institute Import Permit No. 2471). Before conducting bioassays, the IP 46 isolate was host-passaged through laboratory-reared *An. gambiae *s.s. adults in order to maintain its virulence. Conidia were harvested from cultures grown on autoclaved rice substrate (200 g per bag) in nylon bags at 25°C and 12 h photophase, after 15 d incubation. They were then dried in silica gel at 4°C for 4 d. Preparation of stock- and working-solution concentrations formulated in Enerpar oil (Enerpar M002^®^, BP South Africa Ltd) followed standard protocols [4]. Before each experiment, conidia viability (>95% germination on Sabouraud Dextrose Agar) was confirmed. 1200 μl of the working-solution was applied evenly to 15 × 25 cm proofing paper using a metal bar (0.31 mm diameter; paper and applicator bar from RK Print Coat Instruments, London), giving a uniform concentration of 3.3 × 10^6 ^conidia cm^-2^. The treated paper was left to dry for 12 h at 26 ± 1°C and 80 ± 5% RH, and then used to line the inside of plastic exposure tubes (8.2 cm diameter × 12.5 cm height). Untreated control replicates used paper treated with Enerpar oil only.

A total of 30-40 unfed 3-7 d old adult *An. gambiae *s.s. (colony established in 1996, Njage village, Tanzania) or *An. arabiensis *(colony established in 2007, Sagamaganga village, Tanzania) were introduced to the exposure tubes. Four separate bioassays were run (both sexes for each species) and three replicates were carried out for each bioassay. Mosquitoes were held in the tubes for 6 h, after which they were transferred to 9 cm^3 ^holding cages at 26 ± 1°C and 90 ± 5% RH, and provided with 9% glucose/water (w/v) solution. The survival and fungus infection status of mosquitoes were monitored daily for up to 28 d, following procedures described elsewhere [4]. Mosquito survival was analysed by Kaplan-Meier pair-wise comparison and Cox regression analysis, using SPSS version 16. Cox regression generated hazard ratios (HR) indicating the daily risk of dying for a mosquito in each bioassay group.

**Figure 1:**
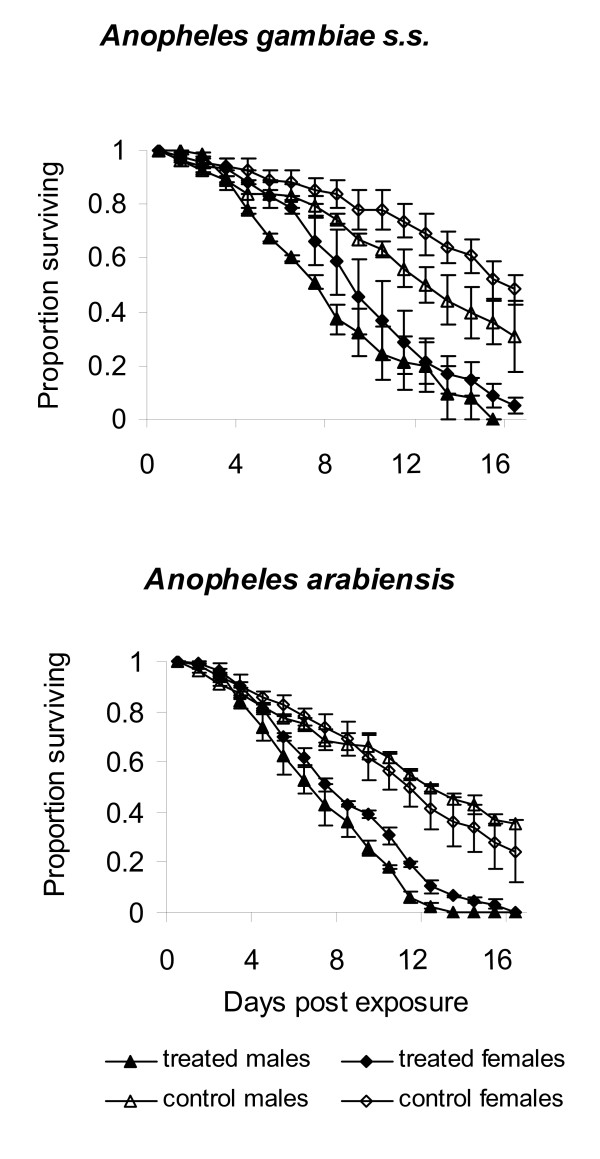
**Survival of adult female and male a) *Anopheles gambiae *s.s. and b) *An. arabiensis *mosquitoes after 6 h exposure to *Metarhizium anisopliae *IP 46 conidia**.

*Metarhizium anisopliae *IP 46 was capable of infecting males and females of both mosquito species: >95% of *An. gambiae *s.s. and *An. arabiensis *cadavers showed fungus sporulation after incubation for 5-6 d. The fungus significantly reduced the survival of all exposed mosquitoes compared to controls (*p *< 0.001, Table 1, Fig. 1); >90% of mosquitoes in the exposure groups had died by day 14 while >25% of control mosquitoes were still alive by this time. All of the control mosquitoes in all bioassays had died by day 28. For *An. gambiae *s.s. the daily risk of dying was over three-fold greater in exposed females (HR = 3.18, *p *< 0.001) and males (HR = 3.81, *p *< 0.001) relative to their controls. A similar trend was observed in exposed females (HR = 2.28, *p *< 0.001) and males (HR = 3.31,* p *< 0.001) of *An. arabiensis*. The daily risk for males was higher than for females in both species (*An. gambiae *s.s. HR = 1.11, *p *= 0.001 and *An. arabiensis *HR = 1.13, *p *= 0.004). Overall, daily risk of dying was higher for exposed *An. gambiae *than *An. arabiensis *(HR = 1.38, *p *< 0.001). The controls for *An. gambiae *survived relatively longer (males MST = 14 d; females MST = 16 d) than those of *An. arabiensis *(male MST = 12 d; female MST = 12 d, Table 1), but this difference was accounted for by Cox regression model which compares relative risks rather than fixed survival time values.

**Table 1:** Pair-wise Kaplan-Meier median survival times (MST) for adult *Anopheles gambiae *s.s. and *An. arabiensis *exposed to oil-formulated *M. anisopliae *IP 46 (treatment) or oil only (control).

Species	Sex	MST ± 1 S.E.		χ^**2 **^value	*p *value
		**Control**	**Treatment**		
				
***An. gambiae *s.s.**	Female	16 ± 0.51	9 ± 0.23	94.58	<0.001
	Male	14 ± 0.76	8 ± 0.30	133.07	<0.001
***An. arabiensis***	Female	12 ± 0.79	8 ± 0.38	63.04	<0.001
	Male	12 ± 0.45	6 ± 0.31	113.13	<0.001

For effective malaria control, entomopathogenic fungi do not need to kill vector mosquitoes instantly [5]. If mosquitoes are able to reproduce and pass genes to the next generation before they are killed by an insecticide the selection pressure for the development of resistance is significantly reduced [6,7]. Here we have shown that the isolate *M. anisopliae *IP 46 kills females of *An. gambiae *s.s. and *An. arabiensis *on average 8-9 d after exposure. By day 14 the majority (>90%) of exposed mosquitoes had been killed. Given that the *Plasmodium *parasite requires approximately 9 to 14 d to infect the mosquito salivary glands, the risk of malaria transmission by fungus-infected mosquitoes is minimal [8]. Similar rates of mortality have been recorded for other entomopathogenic fungi against mosquitoes [4,9-11]. Perhaps most importantly, *M. anisopliae *IP 46 was effective against both *An. arabiensis *and *An. gambiae *s.s. suggesting that it could be used to target both indoor and outdoor resting anophelines. This is the first study demonstrating the susceptibility of adult *An. arabiensis *to *Metarhizium anisopliae*.

Ultimately the success of entomopathogenic fungi against malaria-carrying mosquitoes in any situation may depend on the choice of fungal isolate. This is because of the inter-isolate variation in virulence, spore production and persistence in relation to their ability to withstand sub-optimal environmental conditions [12-16]. The long-standing barriers that have prevented the widespread uptake of biological control agents include low virulence and short-term residual activity. In order to overcome such barriers it is necessary to screen an array of fungal strains to identify those with the greatest potential for development. We found that the isolate *M. anisopliae *IP 46 is able to reduce the survival of adult anophelines within the same time frame as other strains, *M. anisopliae *ICIPE-30 and *B. bassiana *IMI 391510 [4,10,17]. We anticipate that our findings will encourage research into other strains and further investigation and development of IP 46.

## Competing interests

The authors declare that they have no competing interests.

## Authors' contributions

Conceived and designed the experiments: LLM TLR CL. Performed the experiments: LLM DWL INL. Analyzed the data: LLM TLR MJK INL. Wrote the paper: LLM MJK. Reviewed the paper: CL TLR.
